# Model‐Enabled Knowledge Transfer Across Cell Lines, Culture Scales and Conditions

**DOI:** 10.1002/bit.70269

**Published:** 2026-06-16

**Authors:** Luxi Yu, Antonio del Rio Chanona, Cleo Kontoravdi

**Affiliations:** ^1^ Department of Chemical Engineering, South Kensington Campus Imperial College London London UK

**Keywords:** bioprocess modeling, ensemble Kalman filter, knowledge transfer, state estimation

## Abstract

Mechanistic models are central to quantitative understanding and optimization of Chinese hamster ovary (CHO) cell culture processes, but their utility is often restricted by parameter sets calibrated for specific cell lines, scales, or operating conditions. In this study, we present the application of the ensemble Kalman filter (EnKF) to bioprocessing, introducing an ensemble‐based framework for dual state and parameter estimation that enables mechanistic model adaptation across distinct systems. The EnKF recursively assimilates process measurements to update uncertain kinetic parameters and predict system states. This allows a model calibrated for one system to be transferred to another without reparameterization, using only a single experimental dataset. The evolving parameter ensembles provide a time‐resolved sensitivity analysis that identifies which parameters have dominant influence under new process conditions and when their effects become significant. The framework was evaluated across six CHO cell datasets spanning different scales, cell lines, temperatures, and feeding strategies. It accurately reconstructed system dynamics and showed progressive improvement in long‐term predictions as data accumulated. By maintaining full mechanistic transparency while flexibly adapting to new data, the EnKF offers a practical route for knowledge transfer across systems, strengthening the role of mechanistic modeling in data‐informed bioprocess understanding and control.

## 1 Introduction

Effective knowledge transfer across bioprocessing systems is essential for reducing process development timelines, adapting platforms to new cell lines and products, facilitating scale‐up, and optimizing production (Reichelt et al. 2017; Helleckes et al. 2023). However, inherent biological variability and system‐specific differences in metabolism, growth dynamics, and consequently nutrient uptake rates often limit the transferability of process insights across systems (Chen et al. 2019). In the absence of a structured methodology for adopting prior knowledge, process development continues to rely on extensive experimentation, leading to increased cost and longer timelines (Kroll et al. 2017).

Mathematical models offer a systematic framework for understanding and predicting bioprocess behavior through quantitative description of system dynamics (Kiparissides et al. 2011). Mechanistic models, in particular, are widely employed due to their ability to explain biological and physicochemical behaviors based on first principles (Kyriakopoulos et al. 2018; Almquist et al. 2014). While these models can provide valuable insight into cell behavior, their predictive performance is often constrained by system‐specific parameterization (Bayer et al. 2023). When applied to different bioreactor scales, cell lines, or operating regimes, mechanistic models typically require extensive reparametrization to maintain accuracy (Sha et al. 2018).

To overcome these challenges, recent efforts have focused on data‐driven and hybrid modeling approaches designed to improve model transferability. Stand‐alone data‐driven methods, such as transfer learning using artificial neural networks (ANNs) (Rogers et al. 2022) or multi‐fidelity Gaussian process models (Sun et al. 2022; Martens et al. 2025), are flexible and often perform well in data‐scarce regimes (Karimi Alavijeh et al. 2024). However, they typically lack mechanistic interpretability. Hybrid models improve generalizability while partially retaining interpretability by combining mechanistic structure with data‐driven components, as demonstrated in several recent studies (Narayanan et al. 2019; Bayer et al. 2021, 2023; Pinto et al. 2023; Ramos et al. 2024; Cruz‐Bournazou et al. 2022). Yet in many cases, the mechanistic backbone is reduced to mass balances, with core biological behaviors such as metabolic fluxes and growth kinetics captured by black‐box surrogates (Narayanan et al. 2019; Tsopanoglou and Jiménez del Val 2021), reducing the model's capacity to provide mechanistic insight. In other hybrid implementations, system identity is encoded via one‐hot vectors (Helleckes et al. 2024) or embedding layers (Hutter et al. 2021), allowing the model to distinguish between systems based on historical data. However, they do not directly identify the underlying physiological traits responsible for these differences. Moreover, although hybrid models are less data‐intensive than fully data‐driven approaches, they still require multiple datasets to train their data‐driven components (Bayer et al. 2023). This presents a challenge in early‐stage process development, where reliable predictive models are most needed but data is scarce (Lu et al. 2024). In addition, the absence of explicit uncertainty handling increases the risk of overfitting, particularly when datasets are limited or noisy (Schweidtmann et al. 2024).

A fundamental limitation shared across mechanistic, data‐driven, and hybrid models is their reliance on fixed parameter values across process duration. Once parametrized, these models operate deterministically and are typically applied offline, limiting their ability to adapt to deviations in system behavior caused by parameter uncertainty, measurement noise, or biological variability (Hernández Rodríguez et al. 2021; Sinner et al. 2022). This motivates the need for dynamic modeling frameworks that incorporate real‐time measurements to update both states and parameters while retaining mechanistic structure. In this context, state estimation algorithms offer a practical alternative.

Various state estimation techniques have been applied to bioprocess applications, each with distinct advantages and limitations. The extended kalman filter (EKF) remains one of the most widely used methods due to its easy implementation and computational efficiency (Ohadi et al. 2015; Krämer and King 2016; Gopalakrishnan et al. 2011; Narayanan et al. 2020; Dewasme et al. 2019; Bogaerts and Wouwer 2004; Ulonska et al. 2018). EKF linearizes the process model and is generally suitable for mildly nonlinear systems (Lyubenova et al. 2021). However, its accuracy deteriorates when applied to strongly nonlinear and discontinuous processes, which are common in mammalian cell cultures (Yousefi‐Darani, Paquet‐Durand, and Hitzmann 2021). The unscented Kalman filter (UKF) addresses nonlinearities more effectively than the EKF by propagating sigma points through the nonlinear model (Wan and Van der Merwe 2000), with successful applications reported in the bioprocessing literature (Fernandes et al. 2015; Dewasme et al. 2015; Abadli et al. 2021; Krämer and King 2019; Kolås et al. 2009; Yousefi‐Darani, Paquet‐Durand, Hinrichs, et al. 2021; Paquet‐Durand et al. 2022; Kemmer et al. 2023). While more robust than the EKF (Wan and Van der Merwe 2000), the performance of UKF is sensitive to the choice of hyperparameters, often requiring careful tuning to ensure stability and accuracy (Dunik et al. 2012; Scardua and da Cruz 2017). Particle filter (PF) has been applied to many complex bioprocess systems that exhibit highly nonlinear dynamics and non‐Gaussian uncertainty (Golabgir and Herwig 2016; Stelzer et al. 2017; Kager et al. 2019; Sinner et al. 2022; Kager et al. 2018), at the expense of higher computational demand (Elfring et al. 2021). Apart from recursive methods, moving horizon estimation (MHE) formulates the state estimation problem as a constrained optimization over a finite time horizon (Robertson et al. 1996; Rao et al. 2001; Haseltine and Rawlings 2005), explicitly accounts for model constraints and past measurements (Tuveri et al. 2022; Hernández Rodríguez et al. 2021; Alexander et al. 2020). However, the need to solve an optimization problem introduces substantial computational overhead, which can limit its use for real‐time applications.

The ensemble Kalman filter (EnKF) offers a promising solution to many of the challenges associated with state estimation techniques of bioprocessing systems. Originally developed for large‐scale systems in geosciences and reservoir modeling (Evensen et al. 2022), EnKF combines the computational efficiency of Kalman‐based methods with the flexibility of ensemble sampling to handle nonlinearity and uncertainty. Unlike the EKF and UKF, it requires no Jacobian computation or sigma point selection, avoids the high computational burden of PF, and the need to solve an optimization problem in MHE. By propagating an ensemble of model realizations, the EnKF naturally captures the evolution of uncertainty in both states and parameters, without requiring the explicit computation or storage of process covariance matrices (Evensen 2009). This ensemble‐based formulation offers a favorable balance among estimation accuracy, ease of implementation, and computational efficiency.

We have previously applied the EnKF for soft sensing of unmeasured intracellular states in CHO cell cultures (Yu et al. 2023). In this study, we extend its application beyond state estimation to the recursive updating of uncertain kinetic parameters throughout the duration of the culture. By analyzing the temporal evolution of parameter ensembles, we could gain insights into dynamic changes in cellular behavior that would otherwise be masked by static calibration. As the parameter trajectories are continuously refined based on incoming measurements, this approach preserves the mechanistic structure while adapting flexibly to new conditions without the need for reparametrization. This capability supports knowledge transfer by allowing a model developed for one specific system to be applied to different cell lines, scales, or operating conditions using only a single experimental dataset. Moreover, the evolving parameter trajectories enable a form of dynamic sensitivity analysis, where the propagation of ensemble uncertainty highlights which parameters are most influential under the new system conditions.

## 2 Materials and Methods

### 2.1 Mechanistic Model and Experimental Data

#### 2.1.1 Base Mechanistic Model and Calibration Dataset

The mechanistic model used for EnKF implementation was adapted from the cell culture framework described by Kotidis et al. (2019), with model equations provided in Supporting Information A. This reduced model captures key extracellular dynamics, including cell growth and death, metabolism, and antibody synthesis, while excluding intracellular modules related to nucleotide sugar metabolism and glycosylation. The experimental data for this mechanistic model calibration were obtained from fed‐batch cultures of an IgG‐producing CHO‐T127 cell line. The culture was maintained in CD CHO medium (Life Technologies, Paisley, UK) at 36.5°C with 5% ${\;\text{CO}\;}_{2}$ and agitated at 150 rpm. Cells were passaged every 3–4 days, with 50 μM of methionine sulfoximine supplemented during the initial two passages after revival. Cultures were grown in 500 mL vented Erlenmeyer flasks (Corning, Amsterdam, The Netherlands) with a 100 mL working volume and an initial seeding density of $2\times 10^{5}\;{\;\text{cells mL}\;}^{-1}$. 10% (v/v) CD EfficientFeed C C AGT (Life Technologies, Paisley, UK) was added every alternate day starting from day 2 (Kotidis et al. 2019).

To investigate whether the EnKF can adapt a fixed mechanistic model beyond its original parametrization, we applied the method to datasets from diverse bioprocess conditions. These included changes in bioreactor scale, temperature, host cell line, and feeding strategy, as summarized in Table 1. The structure of the model remained unchanged across all cases, with only model parameters updated recursively during the culture.

**Table 1:** Summary of experimental datasets. Only the feed concentration of nutrients considered in the model is reported.

#	Cell line	Bioreactor			Feed concentration (mM)			Reference
		Type	Volume (mL)	Temp (°C)	Glc	Glu	Asn	
1	A	Shake flask	100	36.5	144.4	12.2	27.0	Kotidis et al. (2019)
2	A	Bioreactor	900	36.5	144.4	12.2	27.0	Sou et al. (2015)
3^a^	A	Bioreactor	900	32	144.4	12.2	27.0	Sou et al. (2015)
4	B	Shake flask	50	36.5	144.4	12.2	27.0	Kyriakopoulos and Kontoravdi (2014)
5	B	Shake flask	50	36.5	144.4	21.4	73.6	Kyriakopoulos and Kontoravdi (2014)
6	B	Shake flask	50	36.5	202.2	29.9	103.1	Kyriakopoulos and Kontoravdi (2014)

a Dataset 3 operates at 36.5°C before a downshift to 32°C on day 6.

#### 2.1.2 Same Cell Line: Scale and Temperature Variation

The first three datasets correspond to the same CHO‐T127 cell line (Cell Line A). Dataset 1 represents the original shake‐flask culture used for model calibration, whereas Datasets 2 and 3 were conducted in 1.5 L stirred‐tank bioreactors (DASGIP Technology, Jülich, Germany) to evaluate model transferability across scales and temperature conditions. Both bioreactor datasets were seeded at $8\times 10^{5}\;{\;\text{cells mL}\;}^{-1}$ with a working volume of 0.9 L and maintained at pH 6.9 ± 0.1, 5% ${\;\text{CO}\;}_{2}$, 150 rpm agitation, and active antifoam control. In Dataset 2, the culture temperature was held constant at 36.5°C, while in Dataset 3, a temperature downshift from 36.5°C to 32°C was introduced on day 6. The bioreactor cultures followed the same feeding schedule as the shake‐flask experiment, with 10% (v/v) of Feed C added on even‐numbered days starting on day 2, and were further supplemented with 10% (v/v) of a proprietary nutrient Feed X (MedImmune, Cambridge, UK) (Sou et al. 2015).

#### 2.1.3 Different Cell Line: Feeding Strategy Variation

To evaluate the capability of EnKF to support cross‐cell line knowledge transfer, three additional datasets based on CHO‐GS46 (Cell Line B) were tested, each employing a distinct feeding strategy. Cultures were incubated in 250 mL Erlenmeyer flasks (Corning, Amsterdam, The Netherlands) with a 50 mL working volume, incubated at 36.5°C, 140 rpm, and 8% ${\;\text{CO}\;}_{2}$. Feeding was performed every other day using a tenfold‐concentrated nutrient supplement at 10% (v/v). Dataset 4 employed the same commercial feed used in Cell Line A shake flask culture. Datasets 5 and 6 used in‐house formulations Feed U and Feed U plus 40%, developed from the batch culture nutrient consumption profiles of Cell Line B. Amino acid and glucose concentrations were scaled to sustain a threefold increase in integrated viable cell concentration, with the latter formulation containing 40% higher nutrient levels except for tyrosine, which was limited by solubility. While the feeds contained a broader range of components, only the nutrients represented in the model are listed in Table 1 (Kyriakopoulos and Kontoravdi 2014).

#### 2.1.4 Analytical Methods

Cell concentration and viability were measured either manually using a Neubauer hemocytometer with trypan blue exclusion for shake flask samples or with the automated ViCell system (Beckman Coulter, CA) for bioreactor cultures. Antibody titer in shake flask samples was quantified using the BLItz system with Dip and Read Protein A biosensors (Pall ForteBio, Portsmouth, UK), whereas in the bioreactor studies, secreted mAb concentration was determined by Protein A affinity chromatography. Extracellular glucose, glutamine, glutamate, lactate, and ammonia concentrations were measured using the BioProfile 400 analyzer (NOVA Biomedical, MA) for shake flask experiments, while bioreactor samples were analyzed using the YSI Bioprofiler 800 (NOVA Biomedical, MA). Extracellular amino acid concentrations, including asparagine, were quantified by high‐performance or ultra‐performance liquid chromatography (HPLC/UPLC, Waters, Hertfordshire, UK) using the AccQ‐Tag kit according to the manufacturer's protocol.

### 2.2 EnKF Dual State and Parameter Estimation

**Figure 1:**
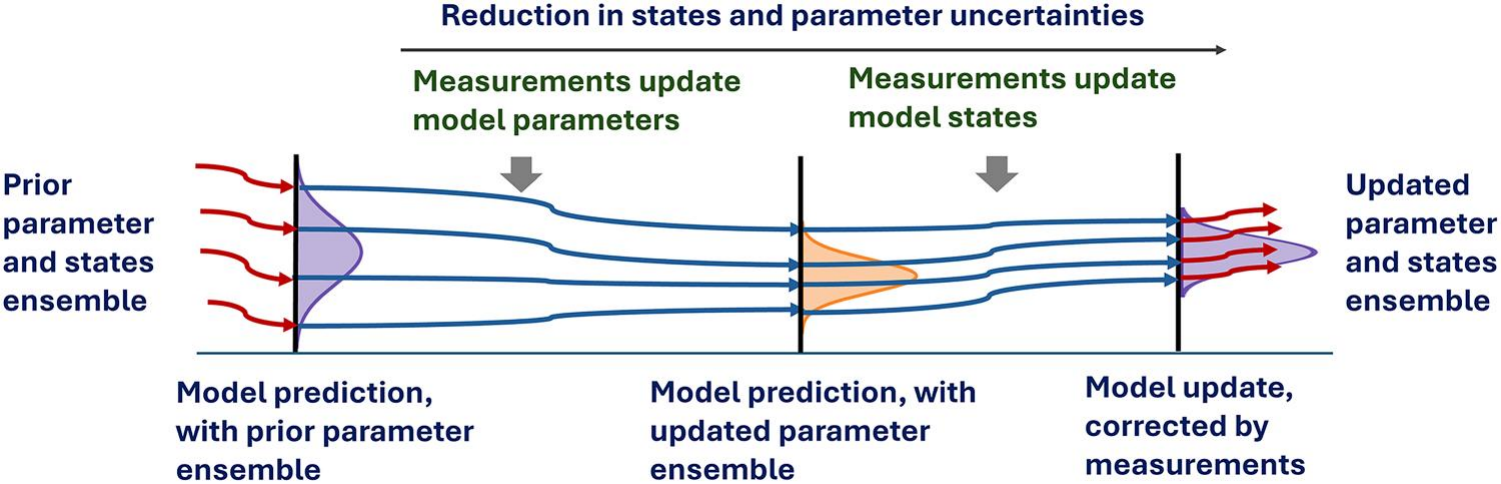
Schematic of dual EnKF framework for state and parameter estimation. State and parameter ensembles are initialized from prior distributions and propagated through the mechanistic model. Upon arrival of new measurements, both ensembles are updated sequentially, progressively refining estimates and reducing uncertainty throughout the culture.

Figure 1 illustrates the structure and information flow of the dual EnKF implementation. At each update step, ensembles of parameters and states, initially sampled from prior distributions, are propagated through the mechanistic model. As new measurements become available, the parameter and state ensembles are sequentially updated, reducing their spread and thereby the associated uncertainty. The narrowing of these distributions reflects convergence towards the true system dynamics, enabling continuous refinement of both parameters and states throughout the culture.

The ensemble is initialized at t=0 with N members, each obtained by independently sampling the state and parameter vectors from Gaussian distributions centered at their prior nominal values, thereby generating N distinct realizations that collectively represent the prior uncertainty:

(1) $$x_{t=0}^{i}∼N\;\left(x_{t=0},P_{t=0}^{xx}\right),\;\theta_{t=0}^{i}∼N\;\left(\theta_{t=0},P_{t=0}^{\theta \theta }\right)$$

where $x_{t}^{i}\in R^{n_{x}}$ is the $n_{x}$‐dimensional state vector and $\theta_{t}^{i}\in R^{n_{\theta }}$ is the $n_{\theta }$‐dimensional parameter vector for the ith ensemble member, with i=1,2,…,N. $P_{t=0}^{xx}\in R^{n_{x}\times n_{x}}$ and $P_{t=0}^{\theta \theta }\in R^{n_{\theta }\times n_{\theta }}$ denote the initial state and parameter covariance matrices, respectively. The observation operator $H\in R^{n_{z}\times n_{x}}$ maps the state vector to the $n_{z}$‐dimensional observation vector $z_{t+1}\in R^{n_{z}}$:

(2) $$z_{t+1}=Hx_{t+1}.$$

In this work, all $n_{x}$ state variables are measured directly, so $n_{z}=n_{x}$ and the observation operator reduces to the identity matrix $H=I_{n_{x}}\in R^{n_{x}\times n_{x}}$. The Kalman gains consequently have dimensions $K_{t+1}^{\theta }\in R^{n_{\theta }\times n_{x}}$ and $K_{t+1}^{x}\in R^{n_{x}\times n_{x}}$. Full‐state measurement is preferable in the knowledge‐transfer setting. In soft sensing, a well‐calibrated model reliably propagates unmeasured states and partial observation suffices. Here, the model operates outside its calibration domain and it is not known a priori which dynamics are misrepresented. Without observing all states, parameter corrections become underdetermined. Full‐state measurement ensures that every model‐prediction mismatch is taken into account in parameter updating.

The mechanistic model used in this study is based on Kotidis et al. (2019), with published parameter values used as the prior mean. The ensemble of prior states evolves through the model using the prior parameter ensemble, with Gaussian‐distributed process noise added to account for model uncertainty:

(3) $$x_{t+1}^{i-}=f\left(x_{t}^{i+},\theta_{t+1}^{i-}\right)+\varepsilon_{t}^{i},\;\varepsilon_{t}^{i}∼N(0,Q)$$

where superscripts − and + denote pre‐ and post‐measurement update estimates respectively. $Q\in R^{n_{x}\times n_{x}}$ is the process noise covariance, and each $x_{t+1}^{i-}$ consists of the model prediction $f\left(x_{t}^{i+},\theta_{t+1}^{i-}\right)$ plus a realization independently sampled from N(0,Q).

The model is numerically integrated with a fine internal time step to capture system dynamics continuously, while measurement updates occur only when experimental data become available, typically once per day in fed‐batch cultures. When off‐line measurements are obtained, an observation ensemble is generated to reflect the associated measurement uncertainty (Evensen et al. 2022):

(4) $$z_{t+1}^{i}=z_{t+1}+\eta_{t+1}^{i},\;\eta_{t+1}^{i}∼N(0,R)$$

where $R\in R^{n_{z}\times n_{z}}$ is the measurement noise covariance, and each $z_{t+1}^{i}$ consists of the measurement vector $z_{t+1}$ plus a realization independently sampled from N(0,R). The experimental error bar can be used as a practical reference for R, accounting for sampling and instrument uncertainty. A lower bound of zero is enforced on each perturbed observation to prevent non‐physical negative concentrations. The measurements are then used to correct the model parameter ensemble. The parameter update follows the standard Kalman correction:

(5) $$\theta_{t+1}^{i+}=\theta_{t+1}^{i-}+K_{t+1}^{\theta }\left(z_{t+1}^{i}-Hx_{t+1}^{i-}\right).$$

The update is scaled by the parameter Kalman gain $K_{t+1}^{\theta }$, which determines how strongly each parameter is adjusted based on the current mismatch between model prediction and measurement.

(6) $$K_{t+1}^{\theta }=P_{t+1}^{\theta x}H^{⊤}{\left(HP_{t+1}^{xx}H^{⊤}+R\right)}^{-1},$$

$P_{t+1}^{\theta x}$ is the cross‐covariance between the parameter and predicted state ensembles, and $P_{t+1}^{xx}$ is the predicted state error covariance, both estimated as sample statistics from the ensemble:

(7) $$P_{t+1}^{\theta x}=\frac{1}{N-1}\sum_{i=1}^{N}\left(\theta_{t+1}^{i-}-{\bar{\theta }}_{t+1}^{-}\right){\left(x_{t+1}^{i-}-{\bar{x}}_{t+1}^{-}\right)}^{⊤},\;P_{t+1}^{xx}=\frac{1}{N-1}\sum_{i=1}^{N}\left(x_{t+1}^{i-}-{\bar{x}}_{t+1}^{-}\right){\left(x_{t+1}^{i-}-{\bar{x}}_{t+1}^{-}\right)}^{⊤}.$$

Here, ${\bar{\theta }}_{t+1}^{-}$ and ${\bar{x}}_{t+1}^{-}$ denote the ensemble means of the predicted parameters and states, respectively. The same perturbed observation ensemble $\left\{z_{t+1}^{i}\right\}$ is then applied a second time to correct the state ensemble. To this end, the states are re‐predicted using the updated parameter ensemble $\left\{\theta_{t+1}^{i+}\right\}$:

(8) $${\tilde{x}}_{t+1}^{i}=f\left(x_{t}^{i+},\theta_{t+1}^{i+}\right)+{\tilde{\varepsilon }}_{t}^{i},\;{\tilde{\varepsilon }}_{t}^{i}∼N(0,Q)$$

where $\tilde{\left(\cdot \right)}$ denotes states and covariances re‐predicted with the updated parameters $\theta_{t+1}^{i+}$, with each ${\tilde{\varepsilon }}_{t}^{i}$ a realization independently sampled from N(0,Q). The corresponding sample covariance is

(9) $${\tilde{P}}_{t+1}^{xx}=\frac{1}{N-1}\sum_{i=1}^{N}\left({\tilde{x}}_{t+1}^{i}-{\bar{\tilde{x}}}_{t+1}\right){\left({\tilde{x}}_{t+1}^{i}-{\bar{\tilde{x}}}_{t+1}\right)}^{⊤},$$

where ${\bar{\tilde{x}}}_{t+1}$ is the ensemble mean of $\left\{{\tilde{x}}_{t+1}^{i}\right\}$. The re‐predicted states are then corrected using the same observation $z_{t+1}^{i}$ via the state Kalman gain $K_{t+1}^{x}$:

(10) $$x_{t+1}^{i+}={\tilde{x}}_{t+1}^{i}+K_{t+1}^{x}\left(z_{t+1}^{i}-H{\tilde{x}}_{t+1}^{i}\right),$$

(11) $$K_{t+1}^{x}={\tilde{P}}_{t+1}^{xx}H^{⊤}{\left(H{\tilde{P}}_{t+1}^{xx}H^{⊤}+R\right)}^{-1}.$$

**Figure 2:**
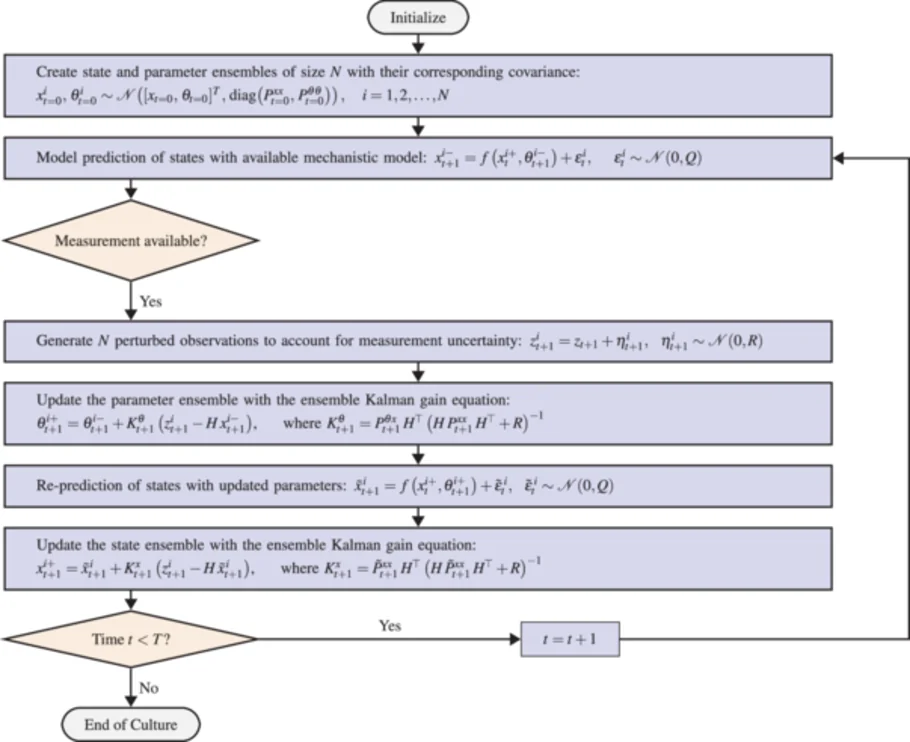
Step‐by‐step workflow of the dual EnKF algorithm, covering ensemble initialization, model‐based prediction, and sequential measurement update via the Kalman gain. The prediction and update cycle repeats at each measurement time point, enabling recursive refinement of both state estimates and model parameters throughout the fed‐batch culture.

In this work, a dual estimation structure was adopted instead of a fully joint formulation in which states and parameters are concatenated into a single augmented vector. In the state update, stochastic process noise is explicitly introduced at each propagation step to capture model uncertainty, effectively inflating the covariance matrix dynamically. For parameters, no artificial noise is injected, ensuring that changes in ensemble spread reflect genuine information gain from experimental data rather than covariance inflation. By maintaining separate ensembles, state propagation remains numerically stable while parameter evolution can retain a relatively large initial covariance to permit adaptive learning. If a large initial covariance were assigned to the augmented state‐parameter vector, the resulting integration would become computationally unstable and inefficient due to the need for repeated numerical integration with overly dispersed trajectories. This separation therefore facilitates both stable computation and biologically interpretable parameter trajectories. The complete sequential update procedure is summarized in Figure 2.

### 2.3 EnKF Uncertainty Quantification

The mechanistic model consists of 8 state variables and 24 kinetic parameters. To reflect the scale and complexity of the system, an ensemble size of 50 was used for the three Cell Line A datasets. For the Cell Line B datasets, a larger ensemble of 75 was selected to account for their greater deviation from the base model and the increased process uncertainty. The rationale for ensemble size selection, including convergence analysis and computational cost, is detailed in Supporting Information B. With the selected ensemble sizes, the total wall‐clock time for simulating an entire fed‐batch culture (12–14 days) is approximately 2–3 minutes on a standard desktop workstation, confirming the feasibility of EnKF for near‐real‐time monitoring and decision support. The accuracy of state and parameter estimation further depends on how uncertainty is represented and propagated through the model. Three sources of uncertainty were specified, namely measurement noise, process noise, and parameter covariance.

#### 2.3.1 Measurement Noise and Process Noise Tuning

Measurement noise covariance R represents observation uncertainty arising from instrumentation, sampling, and handling variability (Evensen 2003, 2009). Initial estimates were based on the standard deviations inferred from experimental error bars. Biological replicates were treated with caution to avoid overestimating measurement noise, which may also reflect process variability. Technical replicates are preferred where available to isolate instrument‐specific error.

Process noise covariance Q accounts for model‐structure limitations, unmodelled dynamics, and residual process disturbances. It was initially estimated from model‐measurement discrepancies and tuned in conjunction with R to regulate the Kalman gain. The ratio between process and measurement noise covariances influences how strongly the filter relies on model predictions versus observed data. This ratio was iteratively adjusted for each dataset to reflect data quality, confidence in model structure, and expected deviations from calibration conditions.

In this work, the model is applied to conditions beyond its original calibration, including different bioreactor scales, cell lines, and feeding strategies. To reflect reduced model confidence under these conditions, the process noise covariance Q is tuned to increase the filter's responsiveness to incoming measurements, ensuring the ensemble is corrected more aggressively toward observed data during adaptation. Final tuned values for R and Q are provided in the Supporting Information C.

#### 2.3.2 Parameter Prior Specification: Design Rationale and Sensitivity

The prior mean parameters are taken directly from Kotidis et al. (2019) and applied identically across all six target datasets without adjustment. To confirm robustness to this choice, all prior means were perturbed by ±10% and ±20% and the filter performance was tested across all datasets. Full results are provided in Supporting Information D.

A common initial parameter covariance matrix was applied across all datasets as reported in Supporting Information E, enabling consistent cross‐dataset comparison of parameter sensitivity. The initial ensemble covariances were set deliberately high but mechanistically plausible, for two complementary reasons. First, a wide ensemble is appropriate at initialization on an uncharacterized target system, where true parameter values are unknown before any measurements are assimilated. Second, a sufficiently large initial covariance prevents premature ensemble collapse and eliminates the need for covariance inflation throughout the run. Conventional EnKF implementations such as those used in geophysical and reservoir modeling routinely apply covariance inflation to counteract collapse from high measurement update frequency (Evensen 2003, 2009). In fed‐batch bioprocessing with a typical run yields only 12–14 measurement updates, ensemble collapse is not a major concern given a sufficiently large initial covariance. Without covariance inflation, any reduction in ensemble spread is attributable solely to measurement assimilation, providing the mechanistic interpretation of which parameters are most constrained by target‐system data. It should be noted that the EnKF parameter covariance differs fundamentally from the static confidence intervals obtained from model calibration, which represent uncertainty estimated from calibration data. Initializing the ensemble from such intervals typically yields insufficient spread for sustained multi‐step updates. As illustrated in Supporting Information F, sensitivity analysis across 0.5×–2.0× the nominal covariance confirms that narrower width remain stable while wider widths introduce instability, placing the reported nominal covariance near the upper practical limit.

To quantify this dynamic spread reduction, the relative decrease in ensemble standard deviation for each parameter over the course of the culture is defined as

(12)

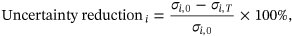

where $\sigma_{i,t}$ denotes the ensemble standard deviation of the ith parameter at time t. This metric serves as a proxy for dynamic sensitivity analysis, indicating which parameters are most constrained by the available data under new process conditions. Parameters showing substantial uncertainty reduction are strongly informed by the data, while those with little or no reduction may be weakly identifiable or insensitive under the given operating regime. Full numerical values for the percentage reduction across parameters are reported in the Supporting Information G.

## 3 Results and Discussion

### 3.1 Model Transfer From Shake Flask to Bioreactor and Temperature Downshift

#### 3.1.1 State Estimation Across Scale and Temperature Shift

**Figure 3:**
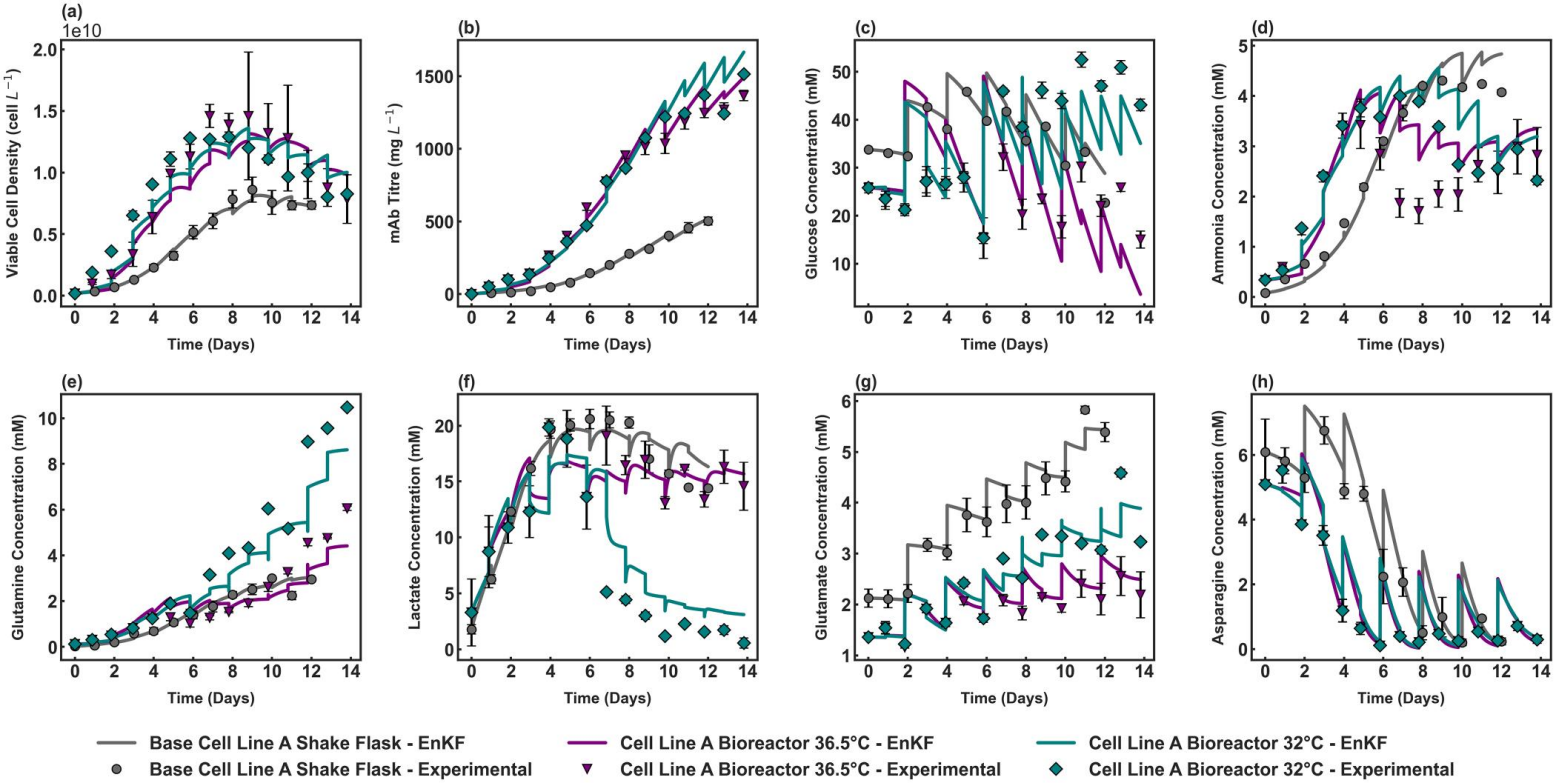
EnKF‐based state estimation for Cell Line A across scale and temperature downshift, including (a) viable cell density, (b) mAb titre, (c) glucose concentration, (d) ammonia concentration, (e) glutamine concentration, (f) lactate concentration, (g) glutamate concentration, (h) asparagine concentration. Trajectories are shown for shake flask at 36.5°C (Dataset 1, gray), bioreactor at 36.5°C (Dataset 2, purple), and bioreactor with temperature downshift to 32°C (Dataset 3, teal). Solid lines show the EnKF posterior mean; markers show experimental measurements (±standard deviation).

The shake flask culture at 36.5°C (Dataset 1, gray) provided the base mechanistic model, which was then adapted to bioreactor cultures at the same temperature (Dataset 2, purple) and following a temperature downshift to 32°C (Dataset 3, teal). The resulting EnKF predictions for all three conditions are shown in Figure 3. The filter facilitates recursive parameter and state updates throughout the culture without model reparameterization.

The higher cell growth and mAb productivity observed at bioreactor scale were both well captured. The EnKF accurately reproduced the overall glucose consumption trends across all three conditions. A slight misalignment was observed under the 32°C condition, which may be attributed to uncertainty in the composition of the proprietary Feed X added in addition to the commercial Feed C. Although Feed X was modeled as part of the nutrient input, its compositional uncertainty was not explicitly represented in the EnKF, meaning its variability was not propagated through the ensemble. Ammonia accumulation plateaued earlier at bioreactor scale compared to shake flask, with the EnKF recovering the general trajectory. Importantly, the filter did not track the ammonia dip between days 6 and 10 for Bioreactor 36.5°C dataset, indicating that it did not overfit to what may represent measurement noise rather than a true metabolic shift.

Following the temperature downshift, glutamine concentrations in the Bioreactor 32°C dataset were substantially elevated, and this was reliably captures by the filter. A distinct shift in lactate metabolism was also observed in response to the temperature downshift, with net lactate consumption occurring after day 6, in contrast to the accumulation observed in shake flask and 36.5°C bioreactor conditions. The lactate consumption trend is successfully captured but the end concentration is slightly lower than that described by the EnKF. Glutamate concentrations varied across datasets, yet the predicted trajectories followed the observed profiles across all three conditions. Asparagine dynamics exhibited clear scale dependence, with the EnKF accurately describing the rate and extent of depletion.

#### 3.1.2 Parameter Evolution for Knowledge Transfer Across Scale and Temperature Shift

**Figure 4:**
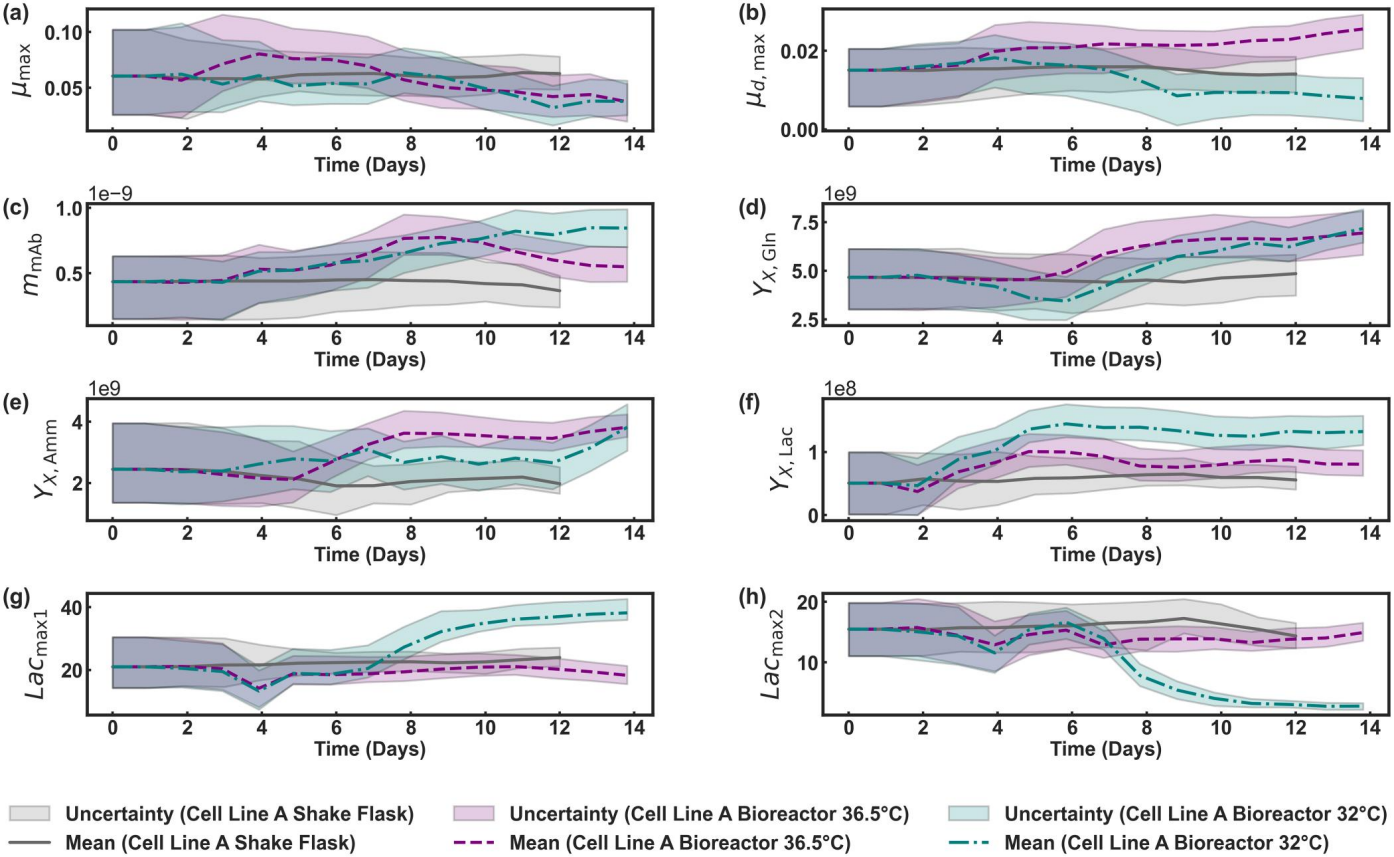
Time evolution of eight estimated model parameters across scale and temperature downshift for Cell Line A, shown for shake flask (Dataset 1, gray), bioreactor at 36.5°C (Dataset 2, purple), and bioreactor with temperature downshift to 32°C (Dataset 3, teal). Parameters shown are (a) maximum specific growth rate ($\mu_{\max}$), (b) maximum specific death rate ($\mu_{\;\text{death,max}\;}$), (c) non‐growth associated mAb production rate ($m_{\;\text{mAb}\;}$), (d) yield of biomass on glutamine ($Y_{X∕Gln}$), (e) yield of biomass on ammonia ($Y_{X∕\mathrm{Amm}}$), (f) yield of biomass on lactate ($Y_{X∕Lac}$), and (g) and (h) lactate consumption activation constants ($Lac_{\max1},Lac_{\max2}$). Solid lines show the posterior mean; shaded bands show the ensemble spread.

Figure 4 shows the time evolution of selected model parameters for Cell Line A across scale and temperature conditions, illustrating how the EnKF progressively adapts the base model to each new operating condition.

*Cell growth and productivity*: Parameter values remain relatively constant across culture duration in the shake flask experiments. This is expected given that this dataset was used for model parameterization and the initial ensemble adequately captures baseline behavior. The trajectory of the maximum specific growth rate $\mu_{\max}$ and death rate $\mu_{d,\max}$ aligns well with the observed viable cell density shown in Figure 3a. In the bioreactor experiments conducted at 36.5°C, $\mu_{\max}$ is increased during early exponential growth, reflecting the higher viable cell densities achieved in this controlled environment with stable pH and dissolved oxygen tension. Interestingly, the trajectory of $\mu_{d,\max}$ shows higher rate of cell death in bioreactors compared to shake flask cultures, potentially due to higher viable cell density achieved. In the 32°C bioreactor, however, a clear reduction in $\mu_{d,\max}$ is observed following the temperature downshift, indicating delayed and slower cell death. This behavior is consistent with the expected benefits of mild hypothermia in CHO cell cultures, which has been shown to prolong viability and extend the productive phase (Sou et al. 2015, 2017). These advantages are also evident in the trajectory of the titer production parameter $m_{mAb}$, which reaches its highest values under 32°C, reflecting enhanced specific productivity with temperature downshift.

*Scale‐dependent glutamine utilization:* The yield of biomass from glutamine $Y_{x,Gln}$ remains relatively constant with time in the shake flasks but increases in both bioreactor cultures from day 6 onwards, reflecting enhanced glutamine utilization for cell growth at larger scale and in a controlled environment. In GS‐CHO cell lines, glutamine is synthesized from glutamate and ammonia via an ATP‐dependent glutamine synthetase reaction (Lin et al. 2019). As ATP is primarily generated through oxidative phosphorylation, the rate of this conversion is sensitive to oxygen availability (Wilson 2017). Improved oxygen transfer in bioreactors supports sustained ATP production, thereby enabling more efficient glutamine synthesis from ammonia. This mechanistic interpretation is further supported by a parallel increase in the yield of biomass from ammonia $Y_{x,\mathrm{Amm}}$. Although glutamine concentration profiles appear similar in the shake flask and 36.5°C bioreactor until day 12 as seen in Figure 3e, the dynamic trajectory of $Y_{x,\mathrm{Gln}}$ inferred by the EnKF reveals scale‐dependent effects as early as day 6.

*Lactate consumption induced by temperature shifts:* The yield of biomass on lactate $Y_{X,\mathrm{Lac}}$ reaches its highest values in the 32°C bioreactor, suggesting increased utilization of lactate as an energy source following the temperature downshift. Scaling up from shake flask to bioreactor at 36.5°C does not substantially alter lactate accumulation, and this is reflected in the relatively stable trajectories of ${\mathrm{Lac}}_{\max1}$ and ${\mathrm{Lac}}_{\max2}$, which remain close to the base model. These parameters represent kinetic constants that regulate the activation of lactate consumption pathways (Kotidis et al. 2019). In the 32°C cultures, ${\mathrm{Lac}}_{\max1}$ increases during the later stages indicating lactate accumulation, while ${\mathrm{Lac}}_{\max2}$ decreases sharply after the downshift due to net lactate consumption. The parameter trajectories provide evidence for the stronger influence of ${\mathrm{Lac}}_{\max2}$ on net lactate consumption, reflecting the bidirectional nature of lactate metabolism.

### 3.2 Model Transfer From Cell‐Line A to B Under Different Feeds

#### 3.2.1 State Estimation Across Cell‐Lines and Feeding Conditions

**Figure 5:**
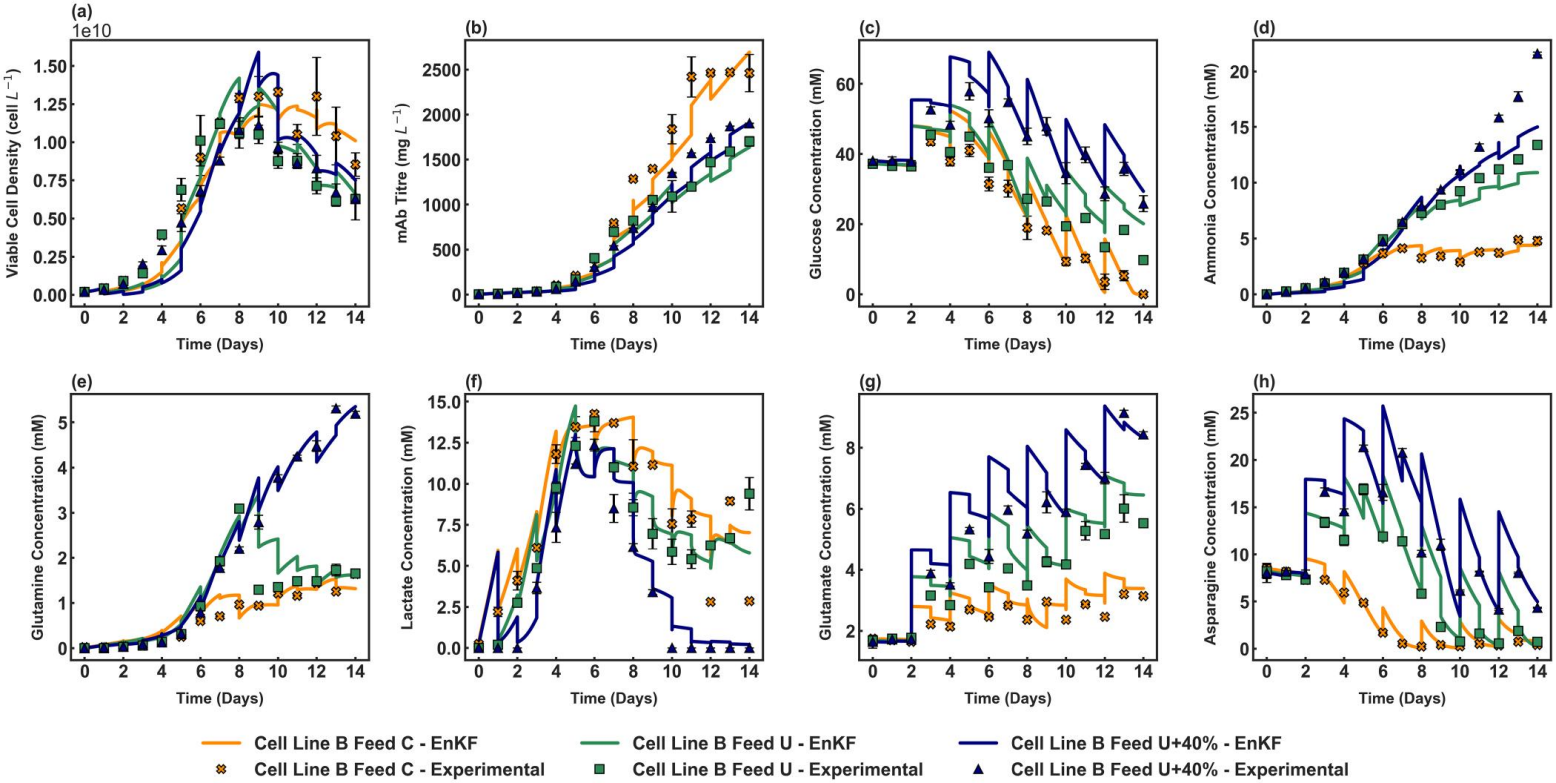
EnKF‐based state estimation for Cell Line B across feeding strategies, including (a) viable cell density, (b) mAb titre, (c) glucose concentration, (d) ammonia concentration, (e) glutamine concentration, (f) lactate concentration, (g) glutamate concentration, (h) asparagine concentration. Trajectories are shown for Feed C (Dataset 4, orange), Feed U (Dataset 5, green), and Feed U plus 40% (Dataset 6, navy), all conducted as shake flask cultures at 36.5°C. Solid lines show the EnKF posterior mean; markers show experimental measurements (±standard deviation). All predictions are initialized from the Cell Line A base model (Dataset 1).

The base model, originally parametrized using Cell Line A shake flask data supplemented with Feed C, was transferred to the Cell Line B. Three feeding strategies were evaluated, Feed C (Dataset 4, orange), Feed U (Dataset 5, green), and Feed U plus 40% (Dataset 6, blue), with all studies conducted in shake flasks. Despite differences in cell line and feed compositions, the EnKF successfully adapted the base model to capture cell growth, productivity, and metabolite dynamics for all three cultures, as shown in Figure 5.

Distinct growth and death profiles were observed across the feeding strategies. Compared to Feed C, Feed U, and Feed U plus 40% both resulted in a steeper decline in viable cell density following peak growth. These differences were accurately captured by the EnKF, even though all predictions were based on the same underlying model structure. The mAb titer profiles were also well reproduced, with Feed C yielding the highest final titer of approximately $2.5\;{\;\text{g L}\;}^{-1}$. This represents a nearly four‐fold increase compared to the baseline model calibrated on Cell Line A, yet the EnKF successfully captured this shift, demonstrating transferability across systems with markedly different productivity levels.

The higher concentrations of glucose and asparagine in Feed U and Feed U plus 40% resulted in different metabolite profiles compared to Feed C. In particular, excess asparagine feeding led to substantial ammonia accumulation, with concentrations exceeding 20 mM by the end of the culture under Feed U plus 40%. While the EnKF described the general trend well, some underprediction was observed during the final stage in this condition. During filter tuning, it was found that a higher ammonia measurement noise was required to stabilize the EnKF in the Feed U plus 40% case. Reducing the measurement noise resulted in a closer fit to the ammonia data but introduced instability at later time points, as the filter was forced too closely towards the measurements and propagated aggressive corrections into correlated states, particularly glutamine, glutamate, and asparagine. The slight mismatch in ammonia is therefore an accepted trade‐off for overall filter stability. Nonetheless, the EnKF well represented the broader dynamics of nitrogen metabolism across conditions, including elevated glutamine and glutamate concentrations in both Feed U and Feed U plus 40%.

Lactate profiles further highlight the flexibility of the EnKF framework. The base model parametrized on the Cell Line A shake flask dataset exhibited net lactate accumulation under Feed C, as shown in Figure 3f. In contrast, Cell Line B culture under the same scale and feeding strategy showed net lactate consumption, indicating this behavior is cell line‐specific. Feed U and Feed U plus 40% resulted in even stronger lactate consumption, with near‐complete depletion by the end of the culture in the latter case. These distinct profiles were accurately described using the same underlying mechanistic model, made possible by the dynamic evolution of the parameter ensemble. Such flexibility would not be attainable under a fixed‐parameter model calibration. Crucially, this adaptability is not achieved at the cost of predictive accuracy. EnKF predictions for Cell Line B under Feed C were benchmarked against a reparametrised open‐loop simulation following Kotidis et al. (Kotidis and Kontoravdi 2019), which first performs global sensitivity analysis (GSA) to identify the most influential model parameters and then re‐fits them specifically for target dataset. This constitutes a rigorous gold standard, but one that is both labor‐intensive and requires considerable modeling expertise to execute for every new system. The EnKF requires neither: no prior sensitivity analysis campaign is needed, and parameter sensitivity emerges naturally through ensemble spread reduction as measurements are assimilated. Despite beginning from Cell Line A base parameters without dataset‐specific fitting, the EnKF achieved accuracy comparable to the reparametrised model. Per‐state quantitative metrics and trajectory comparison are provided in Supporting Information H.

#### 3.2.2 Parameter Evolution for Knowledge Transfer Across Cell‐Lines and Feeding Conditions

**Figure 6:**
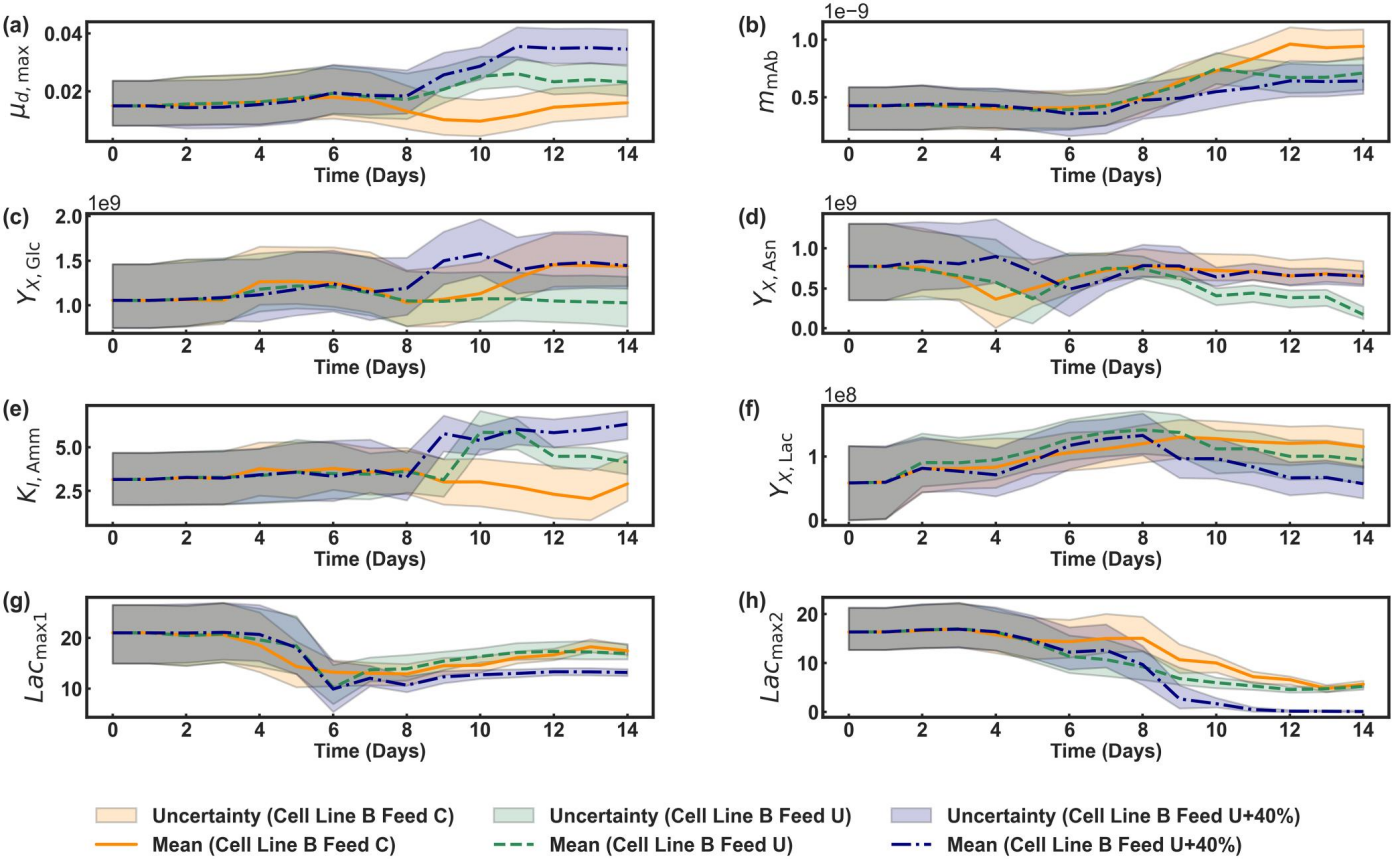
Time evolution of eight estimated model parameters across feeding strategies for Cell Line B, shown for Feed C (Dataset 4, orange), Feed U (Dataset 5, green), and Feed U plus 40% (Dataset 6, navy). Parameters shown are (a) maximum specific death rate ($\mu_{\;\text{death,max}\;}$), (b) non‐growth associated mAb production rate ($m_{\;\text{mAb}\;}$), (c) yield of biomass on glucose ($Y_{X∕Glc}$), (d) yield of biomass on asparagine ($Y_{X∕Asn}$), (e) ammonia inhibition constant ($KI_{\;\text{Amm}\;}$), (f) yield of biomass on lactate ($Y_{X∕Lac}$), and (g) and (h) lactate consumption activation constants ($Lac_{\max1},Lac_{\max2}$). Solid lines show the posterior mean; shaded bands show the ensemble spread.

Figure 6 shows the time evolution of selected model parameters for Cell Line B across feeding strategies, revealing how the EnKF differentiates feed‐specific metabolic behavior.

*Cell growth and productivity:* The trajectory of the maximum specific death rate $\mu_{d,\max}$ provides a clear distinction between the commercial Feed C and the in‐house formulations, Feed U and Feed U plus 40%. Ensemble‐based uncertainty narrows after the exponential growth phase, reflecting the higher sensitivity of $\mu_{d,\max}$ during later stages of cell culture. In Cell Line B supplemented with Feed C, $\mu_{d,\;\text{max}\;}$ remains relatively constant throughout the culture, within the uncertainty of the parameter estimate. In contrast, Feed U shows an increase in the mean death rate during the stationary phase, which is expected given its limited nutrient composition compared to the commercial feed and the associated rise in ammonia levels. Feed U plus 40% exhibits an even higher $\mu_{d,\;\text{max}\;}$, consistent with the pronounced ammonia accumulation observed in this condition and its strong correlation with increased cell death, as illustrated in Figure 5a. Among the three feeding strategies, Feed C supports the highest titer yields, while the two in‐house formulations result in comparable but lower final titers, as shown in Figure 5b. This suggests that improved cell viability under Feed C is accompanied by higher non‐growth‐associated productivity $m_{\mathrm{mAb}}$, contributing to enhanced overall production.

*Effect of excess feeding on biomass yield and nitrogen metabolism:* To assess how elevated glucose and asparagine in Feed U and Feed U plus 40% influence growth, the temporal evolution of biomass yields from glucose $Y_{X,\mathrm{Glc}}$ and asparagine $Y_{X,\mathrm{Asn}}$ was examined. As shown in Figure 6c, $Y_{X,\mathrm{Glc}}$ exhibits broad ensemble spread for all conditions, with only modest changes in the parameter mean over time. This indicates that $Y_{X,\mathrm{Glc}}$ is relatively insensitive to variations in glucose availability and does not strongly influence growth predictions.

In contrast, the $Y_{X,\mathrm{Asn}}$ ensemble spread reduces rapidly, indicating that the filter gains confidence quickly about the contribution of asparagine to biomass formation. $Y_{X,\mathrm{Asn}}$ is initially highest in Feed U plus 40%, reflecting its elevated concentration. Over time, this parameter declines in Feed U with reduced uncertainty, indicating growing model confidence that asparagine contributes less to biomass. Interestingly, $Y_{X,\mathrm{Asn}}$ in Feed U plus 40% drops below that of Feed C by the end, despite significantly higher asparagine availability and uptake rate. This disparity suggests rerouting of excess asparagine through alternative nitrogen pathways. Previous studies have proposed that, under nitrogen‐rich conditions, asparagine synthetase may catalyze a reaction analogous to asparaginase, converting asparagine and glutamate into aspartate and glutamine (Quek et al. 2010). While not conventionally reported in mammalian systems, this reaction has been included in CHO metabolic reconstructions to account for elevated ammonia accumulation under nitrogen‐rich conditions (Carinhas et al. 2013; Kyriakopoulos and Kontoravdi 2014). The parameter trajectory of $Y_{X,\mathrm{Asn}}$ thus offers indirect evidence of such metabolic shifts, revealing insight not captured by concentration data alone.

To further investigate the impact of excess asparagine feeding, the ammonia inhibition parameter $K_{I,\mathrm{Amm}}$ was evaluated. In Feed C, this parameter remains near its initial value with broad uncertainty, consistent with its use in model calibration. In contrast, both in‐house feeds exhibit a sharp increase in $K_{I,\mathrm{Amm}}$ entering stationary phase, accompanied by a rapid drop in uncertainty. This trend aligns with the timing of ammonia accumulation and declining $Y_{X,\mathrm{Asn}}$, indicating that excess nitrogen progressively limits growth in later stages.

Understanding lactate metabolism beyond concentration profiles: To understand how lactate metabolism differs across feeding strategies, the biomass yield on lactate $Y_{X,\mathrm{Lac}}$ and two kinetic parameters ${\mathrm{Lac}}_{\max1}$ and ${\mathrm{Lac}}_{\max2}$ were analyzed. While concentration profiles confirm net lactate consumption under all conditions, they cannot distinguish whether this uptake supports biomass growth or other cellular functions. The parameter trajectories provide a clearer picture. All three conditions show elevated $Y_{X,\mathrm{Lac}}$ early in culture, suggesting enhanced growth‐coupled lactate utilization in Cell Line B. However, only Feed C maintains high yields in later stages, while both in‐house formulations show a decline. This supports the hypothesis that lactate may be increasingly rerouted toward non‐growth‐associated functions such as redox balancing under nutrient‐rich conditions (Morrissey et al. 2026).

The trajectories of the lactate activation constants ${\mathrm{Lac}}_{\max1}$ and ${\mathrm{Lac}}_{\max2}$ further elucidate the dynamics of consumption across feeding strategies. ${\mathrm{Lac}}_{\max2}$ declines across all feeds but most sharply under Feed U plus 40%, in line with complete lactate depletion. In the same condition, ${\mathrm{Lac}}_{\max1}$ also drops steadily, indicating sustained consumption. In contrast, under Feed C and Feed U, ${\mathrm{Lac}}_{\max1}$ rises again, suggesting some lactate accumulation remains despite overall uptake. Importantly, although the concentration profiles indicate net lactate accumulation throughout the early culture phase, the parameter trajectories reveal an earlier underlying metabolic shift. Both ${\mathrm{Lac}}_{\;\text{max}\;1}$ and ${\mathrm{Lac}}_{\;\text{max}\;2}$ begin to decline as early as day 4, well before glucose or amino acid depletion. This behavior mirrors previous observations in Cell Line B batch cultures (Kyriakopoulos et al. 2013) and supports literature findings that lactate consumption can also be triggered by high extracellular lactate rather than nutrient limitation (Zagari et al. 2013; Mulukutla et al. 2012).

The evolution of ${\mathrm{Lac}}_{\max1}$ and ${\mathrm{Lac}}_{\max2}$ across datasets also exemplifies an important consideration in ensemble‐based parameter estimation. Parameters that are jointly informed by available measurements may exhibit posterior correlation, such that their individual contributions cannot be fully resolved independently from data alone. Examining posterior correlations alongside ensemble spread therefore provides a more complete picture of parameter identifiability. A full correlation analysis across all six datasets is provided in Supporting Information I. Despite some degree of correlation, the converged EnKF estimates of ${\mathrm{Lac}}_{\max1}$ and ${\mathrm{Lac}}_{\max2}$ for Cell Line B Feed C are broadly consistent with those reported by the GSA‐guided reparametrised model for this specific dataset (Kotidis and Kontoravdi 2019). Beyond ${\mathrm{Lac}}_{\max1}$ and ${\mathrm{Lac}}_{\max2}$, 7 of the 10 model parameters with the greatest ensemble spread reduction overlap with those identified via GSA by Kotidis and Kontoravdi (2019), despite the EnKF receiving no prior sensitivity information. A detailed parameter‐by‐parameter comparison is provided in Supporting Information J.

### 3.3 EnKF State Estimation Under Data‐Limited Scenarios

#### 3.3.1 Long‐Term Prediction With Real‐Time Measurement Updates

As a culture progresses, measurements become available sequentially and only data collected up to the current time point can be used. At each measurement occasion, the EnKF updates the parameter ensemble and launches an open‐loop simulation forward to harvest. As more data are assimilated, parameter estimates improve and forecasts progressively converge toward the true culture trajectory.

**Figure 7:**
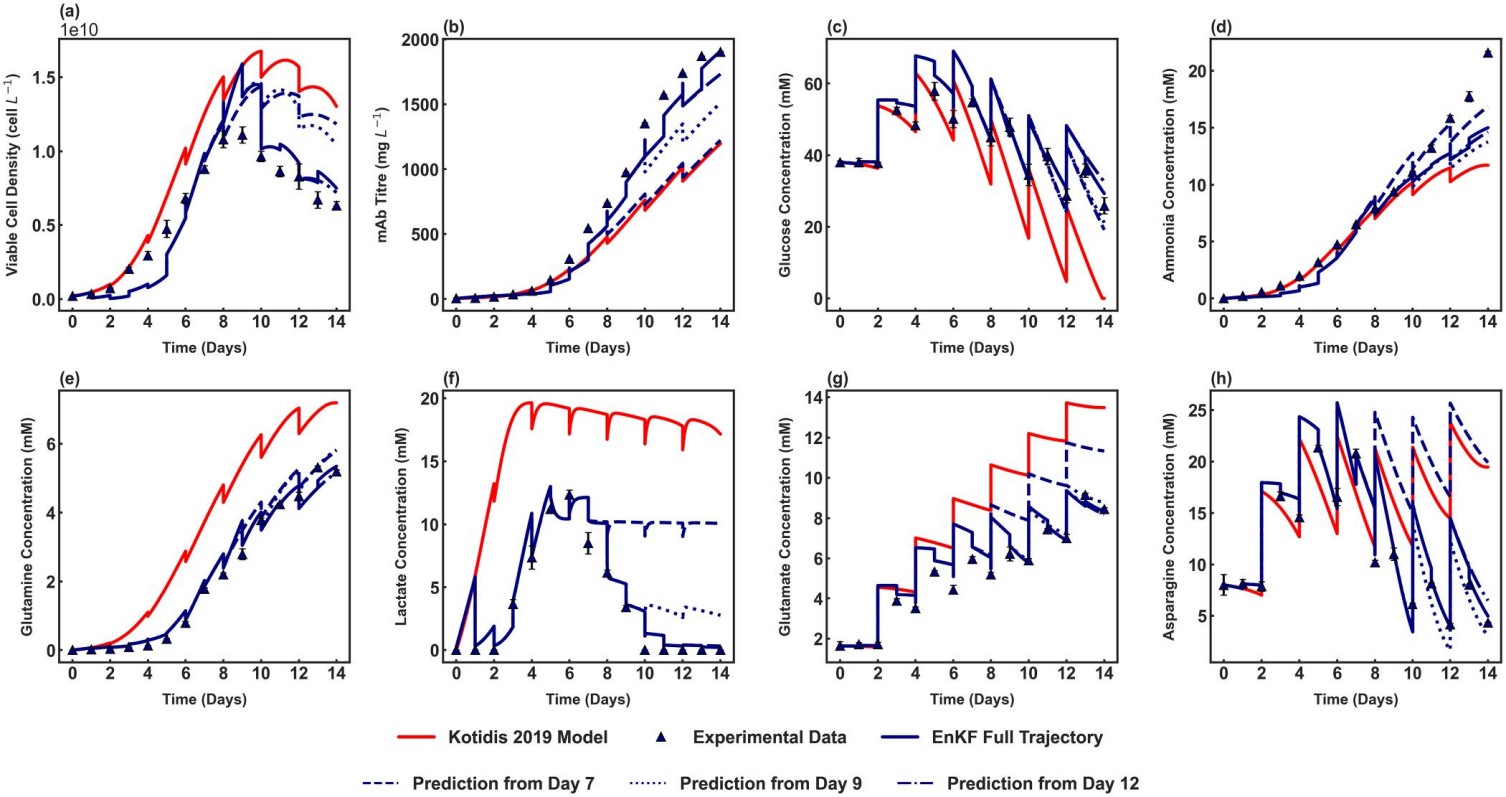
Long‐term EnKF predictions of Cell Line B Feed U plus 40% initiated from day 7 (dashed), day 9 (dotted), and day 12 (dash‐dot) for (a) viable cell density, (b) mAb titre, (c) glucose concentration, (d) ammonia concentration, (e) glutamine concentration, (f) lactate concentration, (g) glutamate concentration, (h) asparagine concentration. EnKF (navy blue) results are compared against experimental measurements and the base Kotidis et al. (2019) model (solid red). Forecasts are open‐loop simulations using the parameter ensemble updated up to each initiation day, with predictions progressively improving over the base model as measurements are assimilated.

Figure 7 illustrates the long‐term prediction performance of EnKF when applied to Cell Line B cultured with Feed U plus 40%, a system that differs significantly from the Cell Line A baseline both in cell line and feeding strategy. By day 7, EnKF predictions already substantially improved upon the baseline mechanistic model for most states, including glucose, glutamine, lactate, and glutamate. By day 9, accurate forecasts are achieved for glucose, glutamine, glutamate, and asparagine. By day 12, all state trajectories align closely with those obtained using the full dataset, confirming the framework's capacity to refine harvest‐day outcomes in real time. The corresponding long‐term prediction results for the remaining datasets are provided in Supporting Information K. Such progressively improving forecasts can support model‐predictive control, where the updated parameter ensemble informs the optimization of future control actions using the most recent process knowledge.

However, the timing at which accurate long‐term prediction becomes possible is case‐dependent. It reflects both the degree of deviation from the base model and the availability of information on key metabolic shifts. For instance, the sharp decline in viable cell density near the end of culture is not reflected in predictions from day 7 or day 9, as this behavior had not yet emerged in the available data. Since the EnKF builds upon a mechanistic model originally developed for a different system, its ability to adapt relies on the progressive assimilation of system‐specific measurements. Thus, while the framework is powerful in adapting to new conditions, its predictive accuracy is fundamentally determined by the timing and availability of incoming data.

#### 3.3.2 State Estimation Under Irregular Measurement Schedules

**Figure 8:**
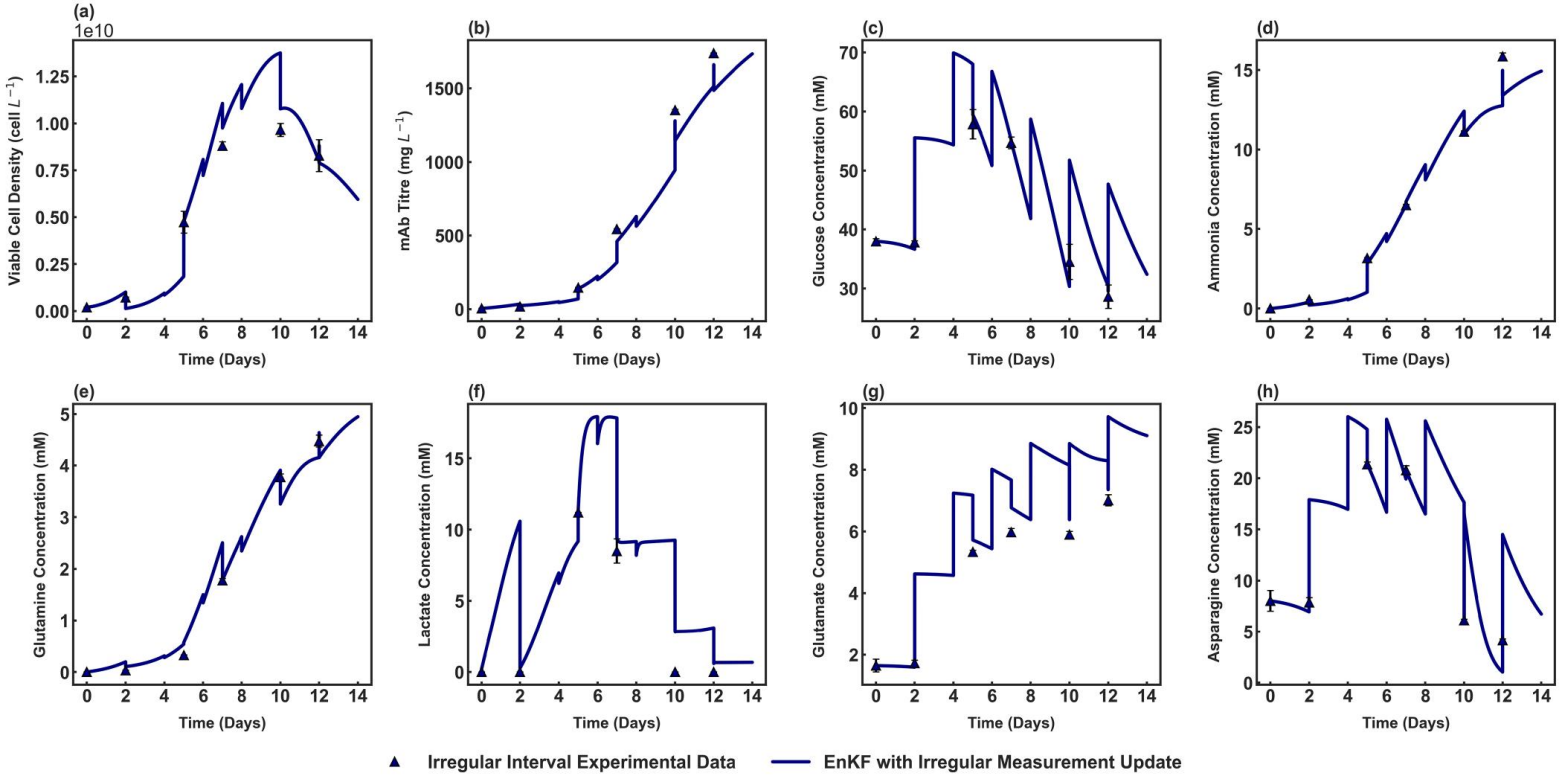
EnKF state estimation of Cell Line B Feed U plus 40% under an irregular measurement schedule of alternating 48‐ and 72‐h sampling intervals for (a) viable cell density, (b) mAb titre, (c) glucose concentration, (d) ammonia concentration, (e) glutamine concentration, (f) lactate concentration, (g) glutamate concentration, (h) asparagine concentration. EnKF trajectories (solid lines) are compared against the irregularly spaced experimental measurements (triangles, ±standard deviation).

In practical bioprocess monitoring, measurements are not always collected at uniform intervals. Equipment downtime, operational constraints, or deliberate sampling strategies can result in irregular or missing time points. To evaluate the robustness of the EnKF framework under such conditions, a scenario was constructed in which measurements are available only at alternating 48‐ and 72‐h intervals, simulating a realistically sparse and non‐uniform sampling schedule. Figure 8 shows the EnKF state estimation results for Cell Line B Feed U plus 40% under this irregular schedule. Despite the reduced measurement frequency and non‐uniform spacing, the EnKF successfully tracks all state variables throughout the culture, maintaining close agreement with the available measurements. The results of the remaining datasets and the quantitative comparison of all datasets are provided in Supporting Information L.

## 4 Conclusions

This work establishes the EnKF as a dynamic and mechanistically grounded framework for knowledge transfer in upstream bioprocess modeling, allowing a system‐specific mechanistic model to be progressively adapted to new conditions without reparameterization. A central contribution lies in the dynamic evolution of parameter ensembles, which provide a temporally resolved view of how the model adapts to the new process. Unlike traditional approaches that treat model parameters as static, the EnKF framework tracks how parameter estimates evolve in response to real‐time measurements. This time‐resolved sensitivity analysis reveals which parameters are the most influential under new conditions, and when their impact on model behavior becomes significant. Importantly, this is achieved without introducing artificial variability through parameter covariance inflation, thereby ensuring that changes in ensemble spread arise from real process data only.

Preserving mechanistic interpretability is particularly valuable for knowledge transfer, as it allows models to explain system behavior beyond predictive performance. While hybrid and black‐box approaches often prioritize generalizability (Rogers et al. 2022; Helleckes et al. 2024; Hutter et al. 2021), recent studies demonstrate that full mechanistic transparency can still be retained across diverse transfer contexts. Richelle et al. demonstrated transfer from fed‐batch to perfusion by simplifying model structure and introducing a “catch‐all” by‐product variable to accommodate changes in operating mode (Richelle et al. 2022). Arndt et al. developed a scale‐up workflow that quantified and compared model parameter distributions across bioreactor volumes, revealing when transfer is valid and when scale‐dependent reparametrization is necessary (Arndt et al. 2021). More recently, Lu et al. incorporated time‐series transcriptomics into a multi‐cell‐line learning (MCL) framework providing enzyme‐level metabolic insight across CHO clones, though requiring high‐performance computing and rich omics datasets (Lu et al. 2024). This work complements these efforts by introducing time‐resolved parameter trajectories, with the EnKF treating biologically meaningful kinetic parameters as dynamic quantities that evolve in response to process measurements. Compared with MCL, the presented method offers a computationally efficient alternative using only routine extracellular measurements, particularly suited for early process development where omics data may not be available.

The ensemble‐based formulation of the EnKF is inherently robust to the large model‐measurement discrepancies that knowledge transfer entails. Conventional estimators such as the EKF and UKF were developed for settings in which the model is already reasonably accurate and corrections remain small. In knowledge transfer, model‐measurement discrepancies may produce large corrections that can drive the explicitly propagated covariance matrix to become ill‐conditioned or indefinite, making these estimators numerically unstable. The EnKF is substantially less susceptible to these failure modes as it represents uncertainty through the ensemble itself rather than an explicitly propagated covariance matrix. The state and parameter covariance is recomputed directly from the ensemble at each update, producing a sample covariance that is positive semi‐definite by construction, with all variances guaranteed to be non‐negative and no Jacobian linearisation required (Evensen 2009).

While this method offers notable advantages in flexibility and interpretability, it relies on two key assumptions. First, the method is designed to capture process‐specific differences through parameter adaptation, without changing the underlying model structure. The six datasets validated here span two CHO cell lines, Cell Line A (CHO‐T127) producing an IgG1 and Cell Line B (CHO‐GS46) producing an IgG4 monoclonal antibody, alongside two bioreactor scales, a temperature downshift, and three feeding strategies. The EnKF framework itself is not restricted to a specific system and can operate with any underlying mechanistic model. The critical requirement is that the model structure adequately represents the key biological phenomena of the target system. The EnKF framework can tolerate some degree of structural imperfection through parameter adaptation, as illustrated by the temperature downshift case. In this dataset, the absence of explicit temperature‐dependent kinetics is compensated through shifts in growth and productivity parameters that remain phenomenologically interpretable. However, when the target system involves phenomena that are structurally absent from the model, the method may still produce state estimates, but the unmodelled dynamics will be distributed across existing parameters in ways that cannot be meaningfully interpreted. Relevant examples include transferring between different expression systems without restructuring the relevant equations, scaling to pilot or manufacturing scale without incorporating mass transfer limitations or other scale‐up effects, or applying the framework to a product modality with substantially different synthesis pathways or kinetics. In such cases, the model structure should be updated to reflect the biology of the new system before the framework is applied. Second, the evolution of parameters is inherently conditioned on the initial ensemble drawn from the base system. In highly nonlinear systems, multiple parameter combinations may yield similar outputs, and the estimated trajectory represents one of the plausible solutions. These assumptions do not undermine the utility of the method but are essential to consider when interpreting biological meaning. Provided the model structure remains appropriate, the dynamic adaptation of parameters still offers valuable insight into system behavior and sensitivity, especially when full reparametrization is not feasible.

As the first demonstration EnKF dual state and parameter estimation in bioprocessing literatures, this work shows how dynamic model adaptation based on routine extracellular measurements can reveal system‐specific behavior without requiring full reparametrization. The resulting dynamic parameter trajectories not only increase the flexibility of transferring mechanistic models across systems, but also provide biologically meaningful insight into process adaptation that extends beyond what is observable from concentration profiles alone. Beyond its methodological contributions, this framework offers a practical and interpretable route for reusing mechanistic models in new bioprocess settings where data are limited and timelines are constrained. From a regulatory perspective, the preservation of mechanistic transparency can support scientific justification for process comparability and risk assessment during tech transfer. In this context, the EnKF establishes itself as a robust and accessible tool for supporting decision‐making across development stages in upstream bioprocessing.

## Author Contributions

**Luxi Yu:** conceptualization, methodology, formal analysis, writing – original draft. **Antonio del Rio Chanona:** conceptualization, supervision, writing – review and editing. **Cleo Kontoravdi:** conceptualization, supervision, writing – review and editing.

## Conflicts of Interest

The authors declare no conflicts of interest.

## Supporting information

Supporting File

## Data Availability

The experimental data and source code supporting the findings of this study are openly available at https://github.com/ly1815/enkf-knowledge-transfer.
